# A Helix Heterodimer in a Lipid Bilayer: Prediction of the Structure of an Integrin Transmembrane Domain via Multiscale Simulations

**DOI:** 10.1016/j.str.2011.07.014

**Published:** 2011-10-12

**Authors:** Antreas C. Kalli, Benjamin A. Hall, Iain D. Campbell, Mark S.P. Sansom

**Affiliations:** 1 Department of Biochemistry, University of Oxford, South Parks Road, Oxford OX1 3QU, UK

## Abstract

Dimerization of transmembrane (TM) α helices of membrane receptors plays a key role in signaling. We show that molecular dynamics simulations yield models of integrin TM helix heterodimers, which agree well with available NMR structures. We use a multiscale simulation approach, combining coarse-grained and subsequent atomistic simulation, to model the dimerization of wild-type (WT) and mutated sequences of the αIIb and β3 integrin TM helices. The WT helices formed a stable, right-handed dimer with the same helix-helix interface as in the published NMR structure (PDB: 2K9J). In contrast, the presence of disruptive mutations perturbed the interface between the helices, altering the conformational stability of the dimer. The αIIb/β3 interface was more flexible than that of, e.g., glycophorin A. This is suggestive of a role for alternative packing modes of the TM helices in transbilayer signaling.

## Graphical abstract

**Graphical Abstract:**

**Figure:**
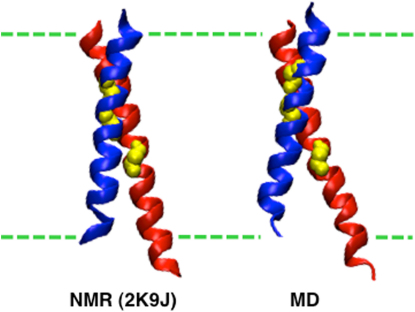

**Highlights:**

► Multiscale simulation have been used to model transmembrane helix heterodimers ► This method is applied to the integrin αIIb/β3 heterodimer ► The model exhibits right-handed packing of the helices, in agreement with NMR structures ► Flexibility of the αIIb/β3 interface suggests of a role for alternative packing modes in transbilayer signaling.

## Introduction

Lateral association of transmembrane (TM) α helices within a lipid bilayer has been studied from a number of perspectives (Bowie, 2005; Langosch and Arkin, 2009; Senes et al., 2004). In particular, TM helix dimerization is important in mechanisms of signaling across membranes by membrane bound receptors, which have ectodomains that bind extracellular ligands and are linked to intracellular signaling domains via single TM helices (Marks et al., 2009). Mutations in TM helices of receptors can perturb signaling and hence result in disease phenotypes (Li and Hristova, 2006). TM helix dimerization also provides a simple model of the lateral association step in membrane protein folding (Bowie, 2005), helping to cast light on the role of simple sequence motifs, such as those involving glycine residues, in providing stable interactions between TM helices (Curran and Engelman, 2003; Javadpour et al., 1999; Russ and Engelman, 2000; Senes et al., 2004).

Molecular dynamics simulations and related computational approaches provide useful tools to model the structure and dynamics of membrane proteins (Khalili-Araghi et al., 2009; Lindahl and Sansom, 2008) and can provide insights into TM helix packing (Psachoulia et al., 2008, 2010). For example, a range of simulation techniques including atomistic and coarse-grained MD simulations (Braun et al., 2004; Cuthbertson et al., 2006; Petrache et al., 2000; Psachoulia et al., 2008; Sengupta and Marrink, 2010), Monte Carlo methods (Vereshaga et al., 2005), potential of mean force calculations (Janosi et al., 2010), and knowledge-based methods (Chugunov et al., 2007) have been used to study the dimerization of TM helices such as glycophorin A (GpA). GpA contains a GxxxG motif in the TM region that is shown to be important for the packing and the stability of GpA homodimer (Anbazhagan and Schneider, 2010; Brosig and Langosch, 1998; Doura and Fleming, 2004; Doura et al., 2004; Russ and Engelman, 1999, 2000).

Integrins provide an important and well-studied example of cell surface receptors whose TM helices are involved in TM signaling (Ginsberg et al., 2005; Harburger and Calderwood, 2009; Hynes, 2002). In mammals, there are 18 different integrin α subunits that may heterodimerize with eight different β subunits to form 24 different integrins. Integrins exist in equilibrium between two states, an inactive low-affinity state and an active high-affinity state (Wegener and Campbell, 2008). Transmembrane helix-helix association plays a crucial role in maintaining the inactive state (Lau et al., 2009; Luo et al., 2004; Schneider and Engelman, 2004; Yang et al., 2009). In the presence of activating proteins such as talin, integrins are believed to activate either by dissociation or by changes in packing of the two TM domains (see, e.g., Anthis et al., 2009; Wegener et al., 2007).

Two recent NMR structures of the αIIbβ3 TM dimer explore the role of the packing of the two integrin TM helices in maintaining the integrin inactive state (Lau et al., 2009; Yang et al., 2009). One structure (pdb id 2K9J; Lau et al., 2009) was obtained in a phospholipid bicelle, the other (pdb id 2KNC; Zhu et al., 2009) in a nonaqueous solvent system. Although showing some differences in the interactions beyond the TM region in the immediate cytoplasmic region of the integrin subunits, the packing of the TM helices is similar in the two structures. In particular, the so-called outer membrane clasp (OMC) region shows close packing of the two helices mediated by a Gx_3_G motif in the αIIb helix. Mutational changes (e.g., αIIbG972L, αIIbG976L/I, and β3G708L/I) in the dimer interface affect dimerization, probably because of induced steric clashes (Berger et al., 2010; Li et al., 2005; Luo et al., 2005).

The integrin TM heterodimer is a more complex system than GpA, and thus provides a valuable test of computational approaches to modeling TM helix dimers of membrane receptors. We have used a multiscale MD approach (combining CG-MD and AT-MD simulations) to explore the αIIb/β3 integrin TM helix heterodimer. We show that simulation methods predict a structure similar to that observed in NMR studies and that mutations suggested to disrupt the dimer perturb the packing of the TM helices.

## Results

### Dimerization of TM Helices by CG-MD

**Figure 1:**
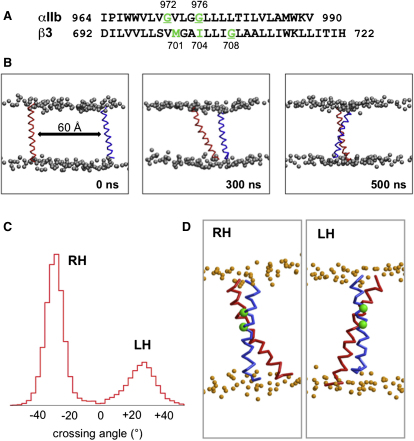
Coarse-Grained Simulations to Self-Assemble an Integrin α/β TM Helix Dimer (A) Sequence of the αIIb and β3 helices used in the simulations. In each sequence key residues at the helix/helix interface are in green and those that have been mutated are underlined. (B) An illustration of the progress of self-assembly of a TM helix dimer by CG-MD simulations. The initial system (0 ns) consists of an αIIb (blue) and a β3 (red) helix in a PC bilayer (shown as the phosphate particles in gray), separated by an interhelix distance of approximately 60 Å. Subsequent snapshots illustrate the helix dimer at 300 and 500 ns. (C and D) Helix crossing angle distribution (C) and representative structures (D) from the CG-WT simulation (see Table 1 for details). A positive value of the crossing angle corresponds to left-handed (LH) helix packing and a negative value to right-handed (RH) packing. The αIIb helix is shown in blue, the β3 helix in red, and the αIIbG972 and αIIbG976 residues in green. The orange spheres represent the phosphate head groups. Note that in The RH helix dimer the αIIb G972 and G976 residues are directed toward the helix interface while in the LH dimer they are directed away from the interface (i.e., toward the viewer).

Our starting point was to use CG-MD simulations to self-assemble integrin TM helix dimers to explore the nature of the predicted helix packing, and, in particular, to define the role of the Gx_3_G motif of the αIIb helix, within the outer membrane clasp (see above). To this end, we started with the sequences of the TM domains of the αIIb and β3 subunits (Figure 1A), as defined in a number of structural studies (Lau et al., 2009). Note that this excludes the nonhelical inner membrane clasp from the C terminus of the αIIb TM helix associated with a GFFKR motif (Lau et al., 2009).

The CG-MD simulations were performed using a high-throughput methodology (Hall et al., 2011), which enabled automatic running of multiple self-assembly simulations, this permitting statistical analysis over an ensemble of approximately 100 structures. In each simulation (Figure 1B) the two helices were initially positioned at a distance of approximately 60 Å away from one another. This separation between the helices is therefore significantly larger than the cutoff distance for electrostatic and van der Waals interactions, and so no interhelix interactions occur at the beginning of the simulation. This ensures that the initial position does not favor dimer formation. During the simulation, the helices diffuse randomly in the bilayer relative to one another, until an encounter leads to formation of a stable dimer occurs, usually within a few hundred nanoseconds (see Figure S5A available online). Visual inspection of the helix dimers reveals them to be largely right-handed (RH), as expected for packing involving a Gx_3_G motif (Russ and Engelman, 2000), although there was a degree of variation in helix packing, both within individual simulations as a function of time and between simulations within the ensemble. This dynamic aspect of TM helix packing can also be seen if one analyses the distribution of helix crossing angles for the whole CG-WT ensemble (Figure 1C); this shows a bimodal distribution, as suggested by preliminary studies (Psachoulia et al., 2010). The major interaction mode (approximately 70% of the structures) corresponds to right-handed packing of the helices, with a mean crossing angle of approximately −30°. We note that this agrees well with the RH packing of the αIIbβ3 TM dimer seen in NMR structures determined in lipid bicelles and in nonaqueous (CH_3_CN/H_2_O) solution (both −27°) (Lau et al., 2009; Yang et al., 2009). The minor mode (30%) corresponding to left-handed (LH) packing of the TM helices was not observed by NMR.

**Figure 2:**
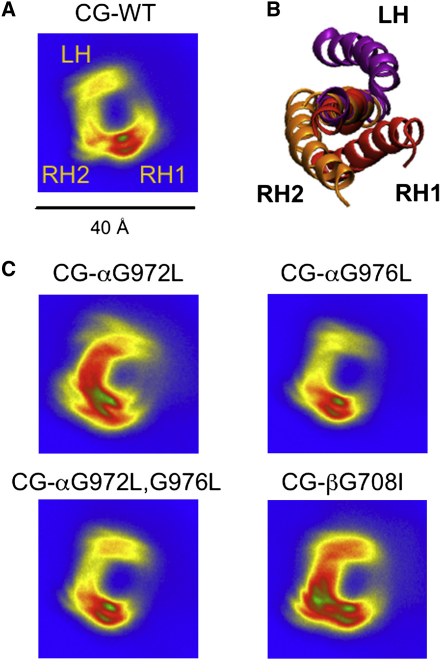
Packing of the Helices in the Dimers Assembled by CG-MD Simulations (A) Spatial distributions of the αIIb helix relative to the β3 helix for the CG-WT simulations. The trajectories for all of the WT CG-MD simulations (see Table 1) were concatenated and the backbone particles of the β3 helix were fitted to a reference structure. The diagram shows the probability density of finding the backbone particles of the αIIb helix at a given point in the bilayer plane around the β3 helix, color-coded such that blue represents low probability through to red then green for a high probability. Three maxima are seen in this distribution, one major (RH1) and two minor (RH2 and LH). (B) Corresponding to the three maxima in (A), three representative structures are shown for the major RH helix dimer (RH1), a minor RH dimer (RH2), and a LH dimer. (C) Spatial distribution analysis for CG simulations in which key glycine residues thought to be at the helix/helix interface are mutated to leucine or isoleucine (see Table 1 for details). In each case, the distribution is altered as the RH1 dimer is disfavored. See also Figures S1, S4, and S5.

Visualization of selected RH and LH structures from the CG-WT ensemble (Figure 1D) reveals a significant difference in the helix/helix interface; in the RH structure, the two glycines of the Gx_3_G motif of αIIb (G972 and G976) are directed toward the β3 helix while in the LH dimer they are directed away from the other helix. To further investigate the packing of the two helices, spatial distributions of the αIIb helix relative to the β3 helix were calculated for the CG-WT simulations. The trajectories for all of the CG-WT simulations (see Table 1) were concatenated and the backbone particles of the β3 helix were fitted to a reference structure in order to calculate the probability density of the backbone particles of the αIIb helix at a given point in the bilayer plane around the β3 helix (Figure 2A). This revealed three maxima, one major (RH1) and two minor (RH2 and LH). These were shown to correspond to three packing modes of the helices, namely, the major RH helix dimer (RH1) as already discussed, a minor RH dimer (RH2), and the LH dimer. To test if the initial position of the helices affects the dimerization of the two helices, similar simulations with the two helices in the same initial positions but with the β3 helix rotated by a 15° increment in each subsequent simulation were performed (approximately 100 simulations). The results were identical (see Figure S5B) with the above simulation (i.e., CG-WT), and therefore for the remaining study we continued using the protocol as described in Experimental Procedures.

In addition to these studies of helical transmembrane regions involving the OMC (Lau et al., 2009), we explored the role of residues in the IMC (see Figure S1). Extending the C terminus of the αIIb subunit by 3 residues to include aromatic residues of the IMC did not greatly perturb the TM helix packing, and a slightly modified RH conformation was observed to be the major form. Extension of the α helix by a further 2 residues to include 2 basic residues resulted in perturbed helix packing and strong interactions with lipid phosphate groups. The loosening of the native packing in the presence of the IMC was also suggested by recent CG simulations using the MARTINI forcefield (Chng and Tan, 2011). Thus, the remainder of this study focused on the TM region as defined in Figure 1.

### Model Assessment and Refinement by Atomistic MD Simulations

**Figure 3:**
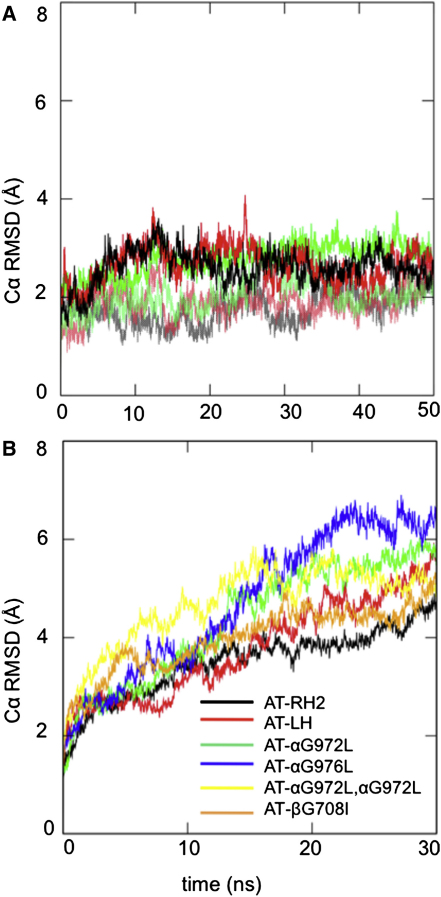
Atomistic Simulations of αIIb/β3 Helix Dimers In each graph, the conformational stability of the α/β helix dimer is analyzed in terms of the Cα root mean square deviation (rmsd) from the initial structure as a function of time. (A) The rmsds are shown for the wild-type helix dimer (the CG-RH1 simulations [pink, pale green, gray] versus the NMR structure [red, green, black]). (B) The rmsds are shown as average across the three repeat simulations for simulations of the mutants and the RH2 and LH models from the wild-type CG simulations (see Table 1 for details). See also Figures S2 and S7.

In order to refine and evaluate further the model(s) of the αIIb/β3 TM helix dimer from the CG-MD self-assembly simulations, the CG structures were converted to full atomistic models, using CG2AT a fragment-based procedure, which has been previously tested for a number of membrane protein systems (Stansfeld and Sansom, 2011). This then allowed us to perform atomistic MD simulations of the models of the TM helix dimer. Such simulations have been used previously to aid in evaluation of models of membrane proteins (Psachoulia et al., 2010; Volynsky et al., 2010). We performed 3 simulations of duration 30 ns for each structure generated by CG2AT (Figure 3; Table 1).

An initial assessment of the conformational stability of the three WT models (RH1, RH2, and LH; see above) was made by comparing their Cα root mean squared deviations from the initial structures in the corresponding simulations (see Figure 3A). From this, it can be seen that the Cα rmsd for the RH1 dimer rapidly reaches a plateau at a value of less than 3 Å, as is generally the case for “stable” membrane protein models (Law et al., 2005). We note that equivalent simulations starting from the NMR structure of the αIIb/β3 TM helix dimer (see more detailed discussion below) yielded a comparable Cα rmsd after 30 ns of approximately 2.5 Å. An additional simulation, using the OPLS forcefield, was performed. This yielded a Cα rmsd of less that 3 Å, characteristic of a stable dimer (Figure S7). In contrast, the Cα rmsds for the RH2 and the LH dimer simulations rise steadily over the course of the simulations, reaching approximately 5 Å for RH2 and approximately 6 Å for LH after 30 ns. This behavior is characteristic of an “unstable” membrane protein model. Similarly, the AT-RH1 simulations yielded a relatively narrow helix crossing angle distribution (see below) characteristic of a stable RH dimer. In contrast, the crossing angle distribution was considerably wider for both the RH2 and LH dimer simulations than for RH1, ranging from −60° to 0° for the RH2 dimer, and from +10° to +60° for the LH dimer, suggesting “looser” helix packing (Figure S6). Previous atomistic MD simulations of the αIIbβ3 NMR structure (i.e., containing the GFFKR motif) also reveal a conformationally stable dimer (Kalli et al., 2011).

### Effects of Mutations Presumed to Be at the Helix/Helix Interface

Various studies have suggested that mutations in glycine residues thought to form the αIIb/β3 TM helix/helix interface (e.g., αIIbG972L, αIIbG976L/I, and β3G708L/I mutations) disrupt helix dimerization (Berger et al., 2010; Li et al., 2005; Luo et al., 2005). We therefore explored the structure and conformational stability of these mutants via multiscale (i.e., CG then AT) simulation.

At the level of CG simulations the relative locations of the two helices in the bilayer plane (Figure 2C) indicate changes in the mode of helix packing in the mutant simulations, that disfavor the RH1 mode that is predominant in WT. For each of the mutant simulations, a CG structure from the favored position in the spatial distribution analysis for each system was converted to an atomistic structure and further (atomistic) MD simulations were initiated (see Table 1 for details). For all of the mutant dimers the Cα rmsd rose quickly to significantly higher values (5–7 Å after 30 ns) than for the AT-RH1 simulations (3 Å). The crossing angle distributions for all the mutant dimer simulations were also wider compared with the WT, ranging from −90° to 0° (Figure S2). Thus, the atomistic simulations confirm the suggestion from CG of weaker helix packing interactions and conformationally less stable dimers in the mutants.

### Comparison with the NMR Structure

**Figure 4:**
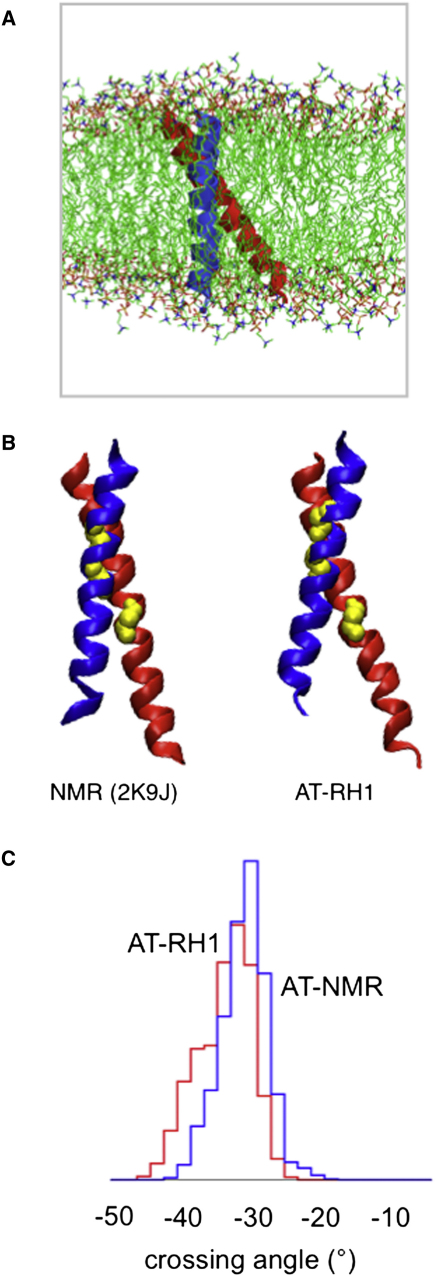
Comparison of the AT-RH1 Structure as Predicted by Simulation in a DPPC Lipid Bilayer with that of the NMR Structure (2K9J; obtained in lipid bicelles (Lau et al., 2009) (A) Snapshot of the AT-RH1 simulation system after CG2AT conversion of the dimer from the corresponding CG-WT simulation. The helices are shown in a DPPC bilayer; water molecules have been omitted for clarity. (B) The NMR (2K9J) and simulated (final frame from one of the AT-RH1 simulations) structures of the αIIb/β3 TM helix dimer compared. The αIIb helix is shown in blue, the β3 helix in red, and the αIIbG972, αIIbG976, and β3G708 residues in yellow. (C) Comparison of the crossing angle distributions from the AT-RH1 (blue) and AT-NMR (red) simulations. Note that the crossing angle of the initial NMR structure (2K9J) was −27°. See also Figure S3.

It is valuable to compare the most stable structure predicted by the simulations (i.e., AT-RH1) to the structures from NMR studies of the αIIb/β3 TM helix dimer, especially the structure solved in a membrane-like environment (2K9J; Lau et al., 2009). The environments in the simulation (a DPPC bilayer in the fluid phase; Figure 4A) and in the NMR studies (a phospholipid bicelle, containing DHPC alongside POPC or DMPC) are similar but nonidentical (Cross et al., 2011; Raschle et al., 2010). In addition to comparing the AT-RH1 simulation with the experimental NMR structure, we also compared it with a simulation (AT-NMR; Table 1) initiated from the NMR structure (of just the residues in the TM helix dimer, i.e., the same residues as in AT-RH1) in a DPPC bilayer.

Comparing one of the final structures from the AT-RH1 simulations with the NMR structure (2K9J) yields a Cα rmsd of 2.2 Å between the simulated dimer and the same (TM helix) region of the NMR structure (see Figure S3). Furthermore, both structures have a crossing angle of −30 ± 3° and a helix/helix interface formed by the same residues (Figure 4B). These crossing angles are consistent with those seen in the NMR (2K9J) ensemble, for which an average value 25° ± 3° is observed.

Both AT-RH1 and AT-NMR simulations yielded a low Cα rmsd from start to end of the simulations (2.5–3 Å; Figure 3A), indicative of a conformationally stable dimer. The crossing angle distributions for the two simulations are very similar (Figure 4C), as are the spatial distribution maps (data not shown). Pairwise comparison of structures from the AT-RH1 and AT-NMR trajectories yielded Cα rmsds of 1–3 Å for the latter halves of each trajectory indicating that both simulations have converged to the same structure within the range of dynamic fluctuations observed in the simulations.

## Discussion

### Biological Implications

The most significant outcome of this study is that the helix/helix interface between the αIIb and β3 integrin TM helices observed in multiscale simulations is the same as that identified in NMR studies (Lau et al., 2009). This demonstrates the predictive capabilities of multiscale MD simulations for identification of helix packing interactions in receptor TM helix dimers even for a heterodimer. It is of particular interest that the correct relative depths of insertion of the two helices were obtained as well as the correct register for the interfacial contacts for the heterodimer.

**Figure 5:**
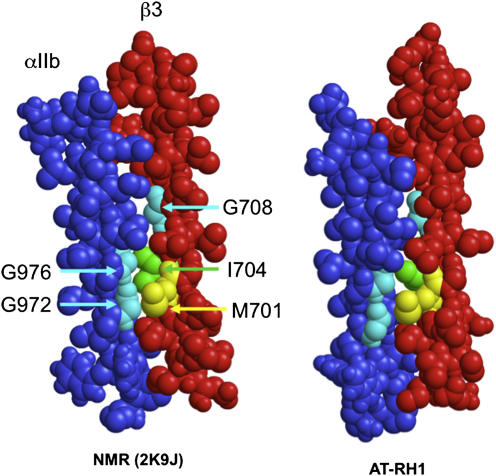
Comparison of the Helix/Helix Interface in the NMR (2K9J) Structure and a Structure from the End of One of the AT-RH1 Simulations The G972,G976 surface (cyan) of the αIIb helix (blue) can bee seen to pack against the M701 (yellow) and I704 (green) side chains of the β3 helix (red). The G708 surface (cyan) of β3 is also seen to pack against αIIb. These key interactions are seen in both the NMR and the simulation structure. See also Figure S6.

The current study has focused on packing of the transmembrane regions of αIIb and β3, and so it mainly refers to interactions involving the “outer membrane clasp,” which includes the Gx_3_G motif of αIIb. The Gx_3_G motif has been extensively studied in the context of helix/helix interactions in membrane proteins (Popot and Engelman, 2000; Russ and Engelman, 2000), especially in the model membrane protein glycophorin A (GpA) (MacKenzie et al., 1997). It should be noted that tight RH helix packing in GpA occurs via a close homodimeric interaction between two identical Gx_3_G motifs. In the integrin dimer the interaction is more complex due to the heteromeric nature of the dimer, with only the αIIb helix donating a Gx_3_G motif. The “exposed” surface on the αIIb helix corresponding to this motif is packed between the bulky hydrophobic side chains of Met701 and Ile704 of β3 (Figure 5) with G708 of β3 packing against the hydrophobic side chain of Leu980 in αIIb. These interactions are largely conserved in other integrin α and β TM helix sequences (Lau et al., 2009), and the results are in good agreement with structure/function analyses of integrin TM helix interactions via mutagenesis studies (Luo et al., 2005) and TOXCAT (Li et al., 2005) and related assays (Berger et al., 2010) of helix/helix interactions in bacterial membrane systems. Besides the αIIb/β3 heterodimer, recent studies (Chng and Tan, 2011; Vararattanavech et al., 2010) for the TM region of the αLβ2 integrin suggest similar packing to that seen in our studies for the helices in the outer membrane clasp (OMC) region (because of the presence of a GxxxG-like motif, i.e., SxxxG). This, in combination with the fact that sequence alignment of the different integrin α subunits reveals the presence of a small-xxx-small motif in many integrin α subunits, suggest that this is a general property of integrins (Vararattanavech et al., 2010). In addition, experimental (Lau et al., 2009; Li et al., 2005; Yang et al., 2009) and computational (Kalli et al., 2011) data suggest that disruption of OMC interactions is a key step in switching integrins from an inactive to an active state. Therefore, the conclusions of this study can be generalized for a variety of integrins and help to explain, in combination with the existing experimental and computational data, the role of the OMC in integrin activation.

The αIIb/β3 interface exhibits a somewhat greater degree of flexibility than the GpA/GpA interface, both in the CG and AT-MD simulations. This is not surprising as GpA forms an exceptionally stable helix/helix dimer, compared with, e.g., receptor tyrosine kinase TM helix dimers (Finger et al., 2009). In contrast, transmembrane signaling for integrins is likely to involve changes in helix packing, possibly via a “scissoring” movement (Kalli et al., 2011). This would be more compatible with a flexible helix/helix interface and/or the existence of alternative packing modes for the TM helices.

We note that alternative modes of TM helix interactions have been observed in NMR structures and discussed in relation to TM signaling by EphA2 receptor tyrosine kinases (Bocharov et al., 2010). This is an aspect of TM helix interaction that merits further pursuit as many widely used assays for such interactions (e.g., the TOXCAT assay; Dews and MacKenzie, 2007; Finger et al., 2009; Li et al., 2004; Russ and Engelman, 1999) may average over the range of possible multiple modes of interaction.

### Simulation Methods and Receptor Structural Biology

Our results demonstrate that a multiscale MD approach can be used to model and characterize the packing and the conformational stability of TM helix dimers. The use of a high-throughput method ensures better sampling in the CG-MD simulations. The subsequent use of atomistic simulations leads to a refined structure with high degree of similarity with the NMR structures, as discussed above. Thus, we combine the strengths of the two methods with CG simulations used for assessing assembly and crossing angles, etc. of TM helices while the more accurate AT simulation are used to assess the relative stabilities of the resultant dimers. This approach is a valuable addition to the ongoing efforts to use computational approaches to model membrane proteins structures and in particular the conformation, dynamics and energetics of helix/helix interactions in the TM domains of receptors.

The integrin heterodimer is a good test case for modeling TM helix dimerization, as it contains a more complex interaction motif than that in GpA or other similar homodimers (e.g., syndecans (Dews and MacKenzie, 2007; Psachoulia et al., 2010). There have been a number of approaches adopted to modeling the integrin TM helix heterodimer (see, e.g., Gottschalk, 2005; Gottschalk and Kessler, 2004 for an early approach) and indeed more recent studies combining modeling and data-based filtering (Metcalf et al., 2009) have shown good agreement with NMR structures.

In the current study, we have shown that a purely simulation-based approach including explicit treatment of the bilayer environment can yield an “NMR accuracy” structure for the integrin TM helix dimer. There have also been recent studies using simulations to select between alternative models (LH and RH) of the EphA2 helix homodimer. In combination, such studies indicate that simulation-based approaches can complement experimental data, providing structural insights into membrane receptor dimers (Jura et al., 2009), and thereby enabling us to establish the link between receptor structure and mechanisms of cellular function.

## Experimental Procedures

### Coarse-Grained MD Simulations

CG simulations used a local modification (Bond et al., 2008) of the MARTINI (Monticelli et al., 2008) forcefield, CG models were constructed for the α-helical regions of αIIb and β3 integrins. These were inserted, parallel and at a distance of approximately 60 Å to one another, in a phospholipid bilayer containing approximately 128 DPPC molecules. After the insertion the N and C termini of the two helices were located in the lipid head group region. The N and C termini of the peptide were not acetylated or amidated. A high-throughput framework was used to run the CG-MD simulations, which allowed approximately 100 simulations of 500 ns each to be performed (Hall et al., 2011). All CG-MD simulations started with the same initial position but with different initial velocities. Analysis for the convergence of the CG simulations suggests that our results converge after using 50% of the simulations for each system (Figure S4). CG-MD simulations were performed using GROMACS (www.gromacs.org) (Lindahl et al., 2001; van der Spoel et al., 2005). The electrostatic/Coulombic interactions were shifted to zero between 0 and 12 Å and the Lenard-Jones interactions between 9 and 12 Å. A Berendsen thermostat (Berendsen et al., 1984) and barostat (reference temperature 323 K and reference pressure 1 bar) was used for temperature (coupling constant 1 ps) and pressure (coupling constant 10 ps) coupling. The integration step was 20 fs.

### From CG to Atomistic Representation

The conversion from CG to atomistic representation was made as described previously (Stansfeld and Sansom, 2011). Briefly, the lipids were constructed by alignment of the coarse-grained particles with energy-minimized atomistic fragments. The CG protein was converted to atomistic using PULCHRA http://cssb.biology.gatech.edu/skolnick/files/PULCHRA/) and MODELER (http://www.salilab.org/modeller/) and then was energy-minimized using the conjugant gradient method.

### Atomistic MD Simulations

After the conversion to atomistic representation three simulations for each system starting from the same initial position but with different initial configurations (i.e., different initial velocities) were performed. The simulations were performed using the GROMOS96 43a1 forcefield (Scott et al., 1999). The LINCS algorithm (Hess et al., 1997) was used to constrain bond lengths and long-range electrostatic were modeled up to 10 Å using the Ewald Particle Mesh (PME). The same cutoff distance was used for the van der Waals interactions. The Parrinello-Rahman barostat and the Berendsen thermostat were used for pressure and temperature coupling. The reference temperature was 323 K. Before every production simulation every system was energy minimized using the conjugant gradient method and subsequently equilibrated with the protein Cα atoms restrained for 0.5 ns (force constant = 1000 kJ/mol/A^2^). Production simulations for 30 ns were performed. The analysis was performed using GROMACS, VMD (Humphrey et al., 1996), and locally written codes.

## Figures and Tables

**Table 1:** Summary of Simulations

Simulation	System	Duration (ns)
CG-WT	αIIb/β3 WT	approximately 100x500
CG-αG972L	αIIb (G972L)/β3	approximately 100x500
CG-αG976L	αIIb (G976L)/β3	approximately 100x500
CG-αG972L,G976L	αIIb (G972L-G976L)/β3	approximately 100x500
CG-βG708I	αIIb /β3 (G708I)	approximately 100x500
AT-RH1	αIIb/β3 WT, RH1 dimer	3x50
AT-RH2	αIIb/β3 WT, RH2 dimer	3x30
AT-LH	αIIb/β3 WT, LH dimer	3x30
AT-αG972L	αIIb (G972L)/β3	3x30
AT-αG976L	αIIb (G976L)/β3	3x30
AT-αG972L,G976L	αIIb (G972L,G976L)/β3	3x30
AT-βG708I	αIIb /β3 (G708I)	3x30
AT-NMR	αIIb /β3 (PDB: 2K9J)	3x50

All simulations used a DPPC lipid bilayer. System sizes ranged from approximately 8500 particles (CG-MD simulations) to approximately 41,500 atoms (atomistic simulations).

## Supplemental Information

Document S1. Seven Figures
