# Supplementary material for: A Helix Heterodimer in a Lipid Bilayer: Prediction of the Structure of an Integrin Transmembrane Domain via Multiscale Simulations
Source: Structure. 2011 Oct 12;19(10):1477–84. doi: 10.1016/j.str.2011.07.014 (PMC3195670; doi:10.1016/j.str.2011.07.014)
Supplement: Document S1. Seven Figures [file mmc1.pdf]

Structure, *19*

Supplemental Information

A Helix Heterodimer in a Lipid Bilayer: Structure Prediction of the Structure of an Integrin Transmembrane Domain via Multiscale Simulations

Antreas C. Kalli, Benjamin A. Hall, Iain D. Campbell, and Mark S.P. Sansom

Inventory of Supplemental Information

Figure S1: It is complimentary to figure 2 and it demonstrates the effect in the packing of the two helices when the sequences were extended towards the integrin inner membrane clasp.
Figure S2: It is complimentary to figure 3 and uses the crossing angle distributions from the AT simulations to compare the stability of the different dimers. The stability of the dimers is also measured in figure 3 using the Cα RMSD.
Figure S3: It is complementary to figure 4 and demonstrates the similarity of the packing of the RH1 mode and the NMR structure by aligning the two structures.
Figure S4: Demonstrated the convergence of the CG-MD simulations which are presented in figure 2.
Figure S5: Demonstrates that the results presented in figure 2 are independent of the initial position of the two helices
Figure S6: It is complimentary to figure 5 and demonstrates the packing of the other two packing modes found in our CG-MD simulations (RH2 and LH). Figure 5 demonstrates only the packing of the RH1 mode in comparison to the NMR structure.
Figure S7: It is complimentary to figure 3 and demonstrates the Cα RMSD of the RH1 simulation using a different forcefield (i.e OPLS). In Figure 3 the Cα RMSD form the same system is shown using the GROMOS forcefield.

A

```
αIIb 964 IPIWWVLVGVLGGLLLLTILVLAMWKV       990
     964 IPIWWVLVGVLGGLLLLTILVLAMWKVGFF    993
     964 IPIWWVLVGVLGGLLLLTILVLAMWKVGFFKR  995
β3   692 DILVVLLSVMGAILLIGLAAALLIWKLLITIH 722
```

(In the printed sequences, "GFF" and "GFFKR" are underlined.)

B

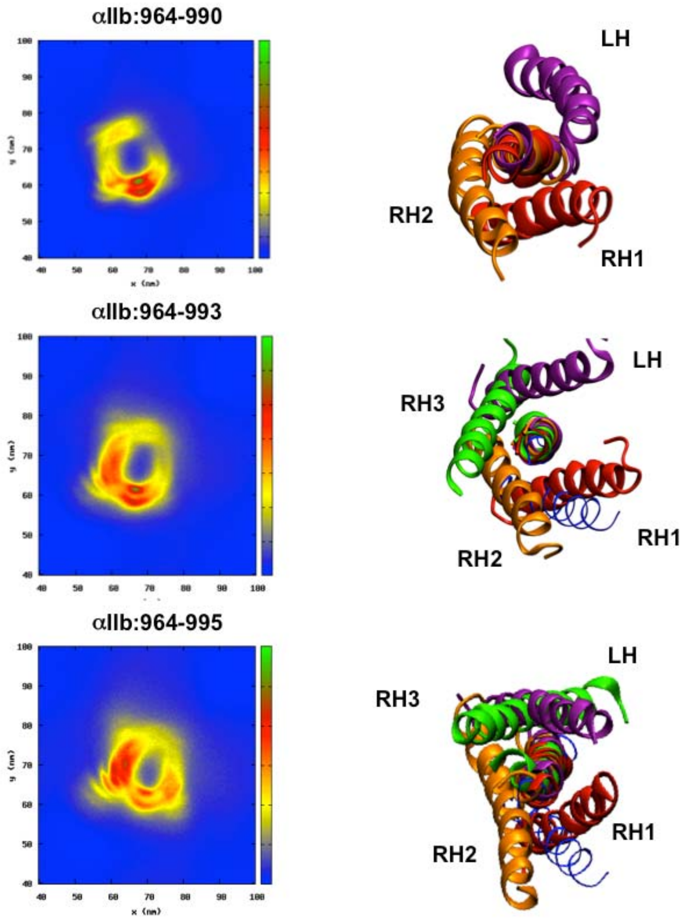

Figure S1, related to Figure 2 :

A. Sequences used in the simulations. The sequence of the TM helix of the αIIb subunit (residues 964-990) is shown as well as the two extended αIIb sequences (964-993 and 964-995; see main text for details).

B. Spatial distributions of the αIIb helix relative to the β3 helix for the CG-WT simulations using the αIIb TM helix (964-990) and using the extended (i.e. 964-993 and 964-995) αIIb helices. Other details are as for Fig. 2 of the main text.

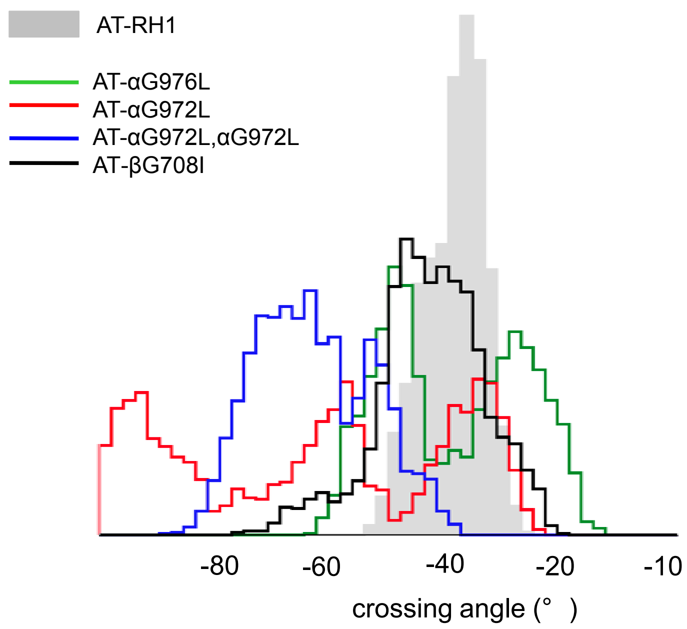

Figure S2, related to Figure 3:
Comparison of the crossing angle distributions from the AT-RH1 wild type (grey filled histogram) and for the AT simulations of various mutants (coloured lines as labelled). The crossing angle was calculated using the last 15 ns of all simulations.

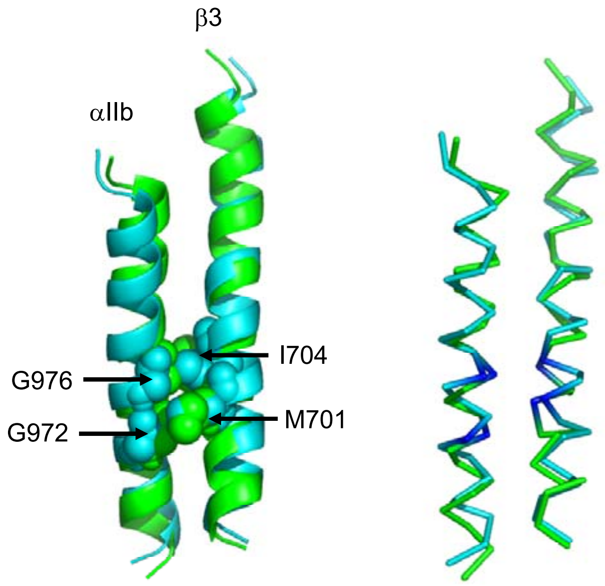

Figure S3, related to Figure 4:
Comparison of the helix/helix interface of structures from the end of one of the AT-NMR (green) and one of the AT-RH1 (cyan) simulations. The Cα RMSD between the two structures is 2.2 Å. The G972 & G976 residues of the αIIb helix and the M701 & I704 residues of the β3 helix are indicated.

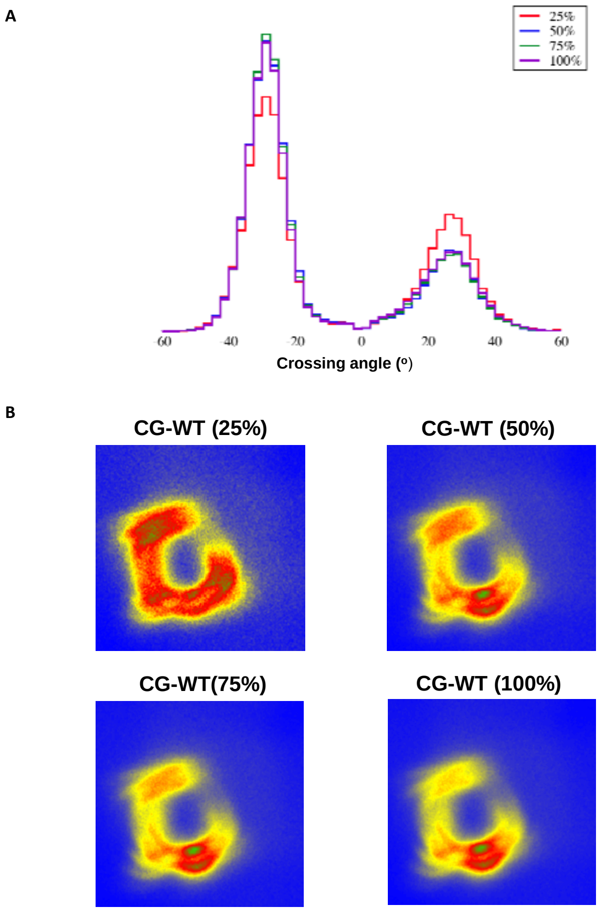

Figure S4, related to Figure 2:

Convergence analysis. The crossing angle distribution (A) and the spatial distribution of the αIIb helix (B) form the CG-WT simulations is shown by using 25%, 50%, 75% and 100% of the simulations. The systems used for the 25%, 50% and 75% subsets were chosen randomly. This analysis suggests that the simulation converges after 50%.

A

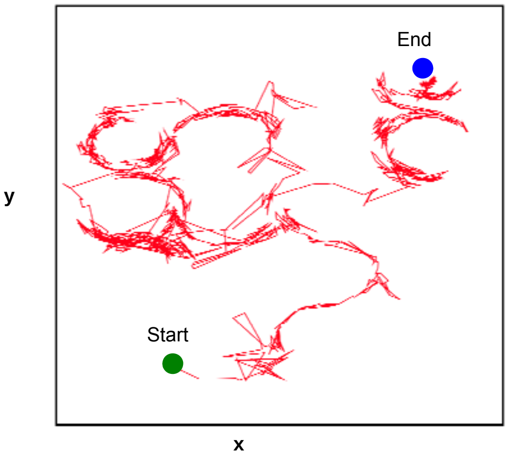

B

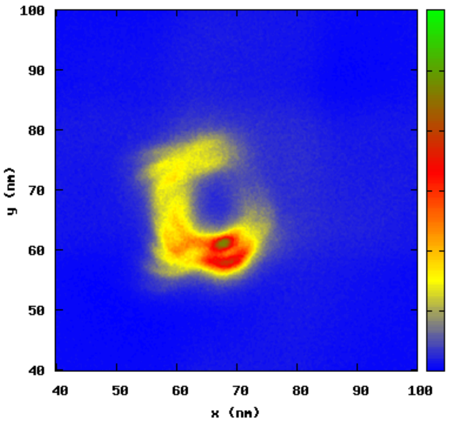

Figure S5, related to Figure 2:

A.Example of the motion of the αIIb helix relative to the β3 helix in the plane of the lipid bilayer. The position of the center of mass of the αIIb helix from the CG-WT simulation is shown. The β3 helix is fixed in the xy plane. The coordinates (x,y) of the mobile helix are drawn in red to show the dimerization of the two helices. The starting position of the αIIb helix is shown in green and the final in blue.

B. Spatial distributions of the αIIb helix relative to the β3 helix for the WT sequence with the β3 helix successively rotated by 15° at the beginning of each simulation. Again ~100 simulations were performed. The spatial distribution is identical with that from the WT-CG simulation.

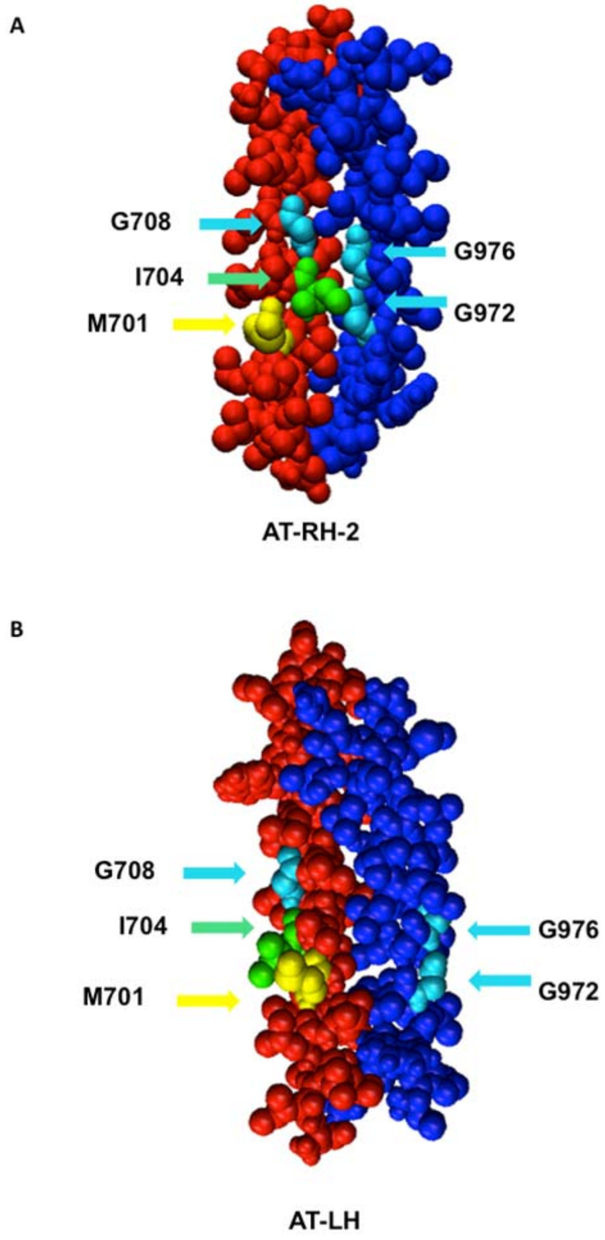

Figure S6, related to Figure 5:
Helix/helix interface from a structure from the end of one of A the AT-RH2 and **B** the AT-LH simulations. The G972,G976 surface (cyan) of the αIIb helix (blue) can be seen to pack away from the M701 (yellow) and I704 (green) sidechains of the β3 helix (red). The G708 surface is shown in cyan. These key interactions are seen in both the NMR and the AT-RH1 simulation structure.

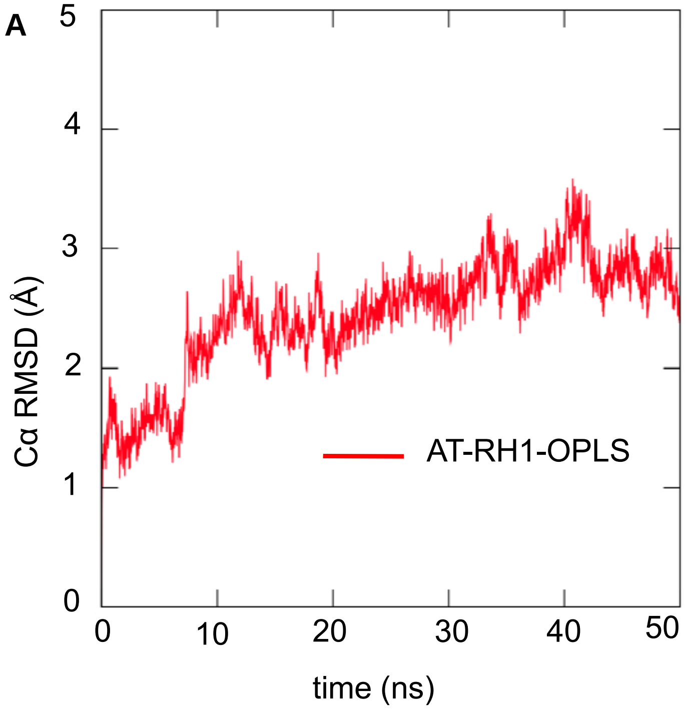

Figure S7, related to Figure 3:
Conformational stability of the α/β helix dimer from an AT-RH1 simulation using the OPLS forcefield as analysed in terms of the Cα root mean square deviation (RMSD) from the initial structure as a function of time. The RMSD was calculated using the αIIb 966-988 and β 694-720 residues.
